# The toxic effects of α-hemolysin and β-hemolysin produced by *Staphylococcus aureus*

**DOI:** 10.3389/fimmu.2026.1811267

**Published:** 2026-05-22

**Authors:** Qiuyu Zhang, Yu Wu, Dan Luo, Fengshan Fang

**Affiliations:** 1 Medical Laboratory Center, Pu’er People’s Hospital, Pu’er, China; 2 Tumor Center, Pu’er People’s Hospital, Pu’er, China

**Keywords:** cell death, inflammatory regulation, *Staphylococcus aureus*, α hemolysin, β hemolysin

## Abstract

*Staphylococcus aureus* (*S. aureus*) is a Gram-positive bacterium widely distributed in nature, which can cause skin and soft tissue infections, sepsis, pneumonia and other invasive diseases. In recent years, the emergence and spread of drug-resistant strains such as *Methicillin-resistant Staphylococcus aureus* (*MRSA*) and *Vancomycin-resistant Staphylococcus aureus* (*VRSA*) have greatly increased the difficulty of clinical treatment, and there is currently no effective vaccine for the treatment of *S. aureus*. The pathogenicity of *S. aureus* depends on a complex and diverse set of virulence factor systems. Alpha hemolysin and beta hemolysin are important toxins produced by it. This article summarizes the toxic effects of α hemolysin and β hemolysin and better understanding the roles of α hemolysin and β hemolysin in the infection process of *S. aureus* can provide a reference for exploring the treatment of *S. aureus* with antitoxins.

## 1 Background

*Staphylococcus aureus* (*S. aureus*) is one of the most important and lethal Gram-positive bacteria in clinical practice, posing a serious threat to global public health (1, 2). As a classic zoonotic pathogen, *S. aureus* is capable of causing a broad spectrum of clinical infections, including common skin and soft tissue infections, as well as life-threatening conditions such as necrotizing pneumonia, osteomyelitis, infective endocarditis, and sepsis (3–5).

To date, there is no effective vaccine against *S. aureus*. Current clinical management of *S. aureus* infections relies primarily on antibiotics. However, with the global spread of multi-drug resistant strains such as *Methicillin-resistant Staphylococcus aureus* (*MRSA*) and *Vancomycin-resistant Staphylococcus aureus* (*VRSA*), the failure rates of conventional antimicrobial therapies have risen significantly (6, 7). Therefore, this underscores the urgent need to develop novel therapeutic approaches. Research has demonstrated that the pathogenicity of *S. aureus* is not solely dependent on the survival of the bacteria but is driven by a complex network of virulence factors such as hemolysins and enterotoxins secreted by the bacteria (8). Thus, targeting virulence factors represents a novel therapeutic strategy, providing a crucial breakthrough for addressing infections caused by drug-resistant bacteria. Among these potential anti-toxin targets, hemolysins occupy a prominent position due to their widespread presence in clinical strains and their direct contribution to host tissue injury. The hemolysin family consists of four major types: α, β, γ, and δ. Among them, α-hemolysin, as a potent pore-forming toxin, is primarily responsible for acute tissue necrosis. Whereas β-hemolysin, through its unique sphingomyelinase activity, plays a key role in immune evasion.

Accordingly, this review will focus on these two representative toxins, elaborating on their molecular pathogenic mechanisms and toxic effects, with the aim of providing a theoretical foundation for the development of novel anti-*S. aureus* agents.

## 2 α-hemolysin

Hla is a water-soluble 33KDa monomer secreted by bacteria, encoded by the Hla gene. Seven alpha hemolysin monomers form a non-dissociated heptamer precursor pore (9). Subsequently, the seven subunits insert into the lipid bilayer, forming a cylindrical transmembrane pore (10, 11), thereby causing uncontrolled ion exchange between the inside and outside of the cell (12).

The Hla receptors currently reported mainly fall into two categories. The first category is non-specific lipid receptors: at higher concentrations of Hla, Hla can directly bind to the lipids of the plasma membrane (PM). The crystal structure of the Hla dimer complex with glycerophosphocholine (GPC) and di-acyl phosphatidylcholine (DiC3PC) indicates that Hla can directly interact with the lipid head group through the gap between the marginal and stem domains (13). There is evidence suggesting that phosphatidylcholine (PC) may be a receptor of Hla (14). Sphingomyelin (SM) and PC can form lipid rafts with cholesterol (15), and this micro-structural domain can act as a concentration platform for membrane-associated proteins, thus possibly mediating the rapid oligomerization of pore toxins (14, 16).

The second category is protein receptors ADAM10: ADAM10 is a type I transmembrane protein that is widely expressed in mammalian cells and has over 40 substrates (17, 18). It was first identified as a high-affinity receptor for Hla in 2010 and is crucial for mediating its cytotoxicity under low Hla concentrations (19). Firstly, ADAM10 exists in multiple functional forms. It is initially synthesized as an inactive proenzyme of approximately 90 kDa (Pro-ADAM10), which needs to be cleaved by proprotein convertases (such as Furin or PC7) to remove the leader peptide before it can be converted into a mature active form of approximately 68 kDa. Notably, this maturation process is not the end. The TspanC8 family (particularly Tspan33) acts as a key chaperone protein, not only assisting in the export of ADAM10 from the endoplasmic reticulum but also determining its substrate specificity (20).

Secondly, in highly polarized epithelial cells, mature ADAM10 is not evenly distributed on the cell membrane but is “anchored” at the intercellular junctions through Tspan33. Subsequently, its intracellular tail binds to Afadin and is “locked” in place. Eventually, under the scaffold effect of the PLEKHA7-PDZD11 complex, a high-density receptor cluster is formed. This spatial aggregation is crucial for Hla to exert its highly efficient toxicity - it confines the receptors to specific microdomains, promoting the oligomerization of the toxin and the formation of stable pores; conversely, if this complex is disrupted, ADAM10 will disperse to the lateral membrane, and the toxin pores are easily endocytosed and cleared, allowing the cell to survive (21).

Finally, ADAM10 exhibits significant tissue and cell specificity. Although it is widely expressed in immune cells and endothelial cells, in epithelial tissues, its expression level is negatively correlated with the cell differentiation status: it is highly expressed in the trachea and bronchi but enriched in the basal layer of the epidermis (undifferentiated cells), and decreases as cells differentiate upwards (22, 23).

### 2.1 The toxic effect of Hla

#### 2.1.1 Cell death and inflammatory storm

Hla can interfere with the functions of immune cells by reshaping the cell death spectrum. It alternately employs the “utilization - destruction” strategies at different stages of infection.

At the level of innate immunity, the interaction between *S. aureus* and macrophages exhibits a bidirectional mechanism. In the early stage of infection or when the toxin level is low, Hla can activate the NF-κB signaling pathway, thereby upregulating the expression of the anti-apoptotic gene MCL-1 (myeloid cell leukemia factor-1), thereby inhibiting macrophage apoptosis (24). This provides an ecological niche for *S. aureus* to survive within macrophages, allowing it to evade the clearance of immune effectors and proliferate. As the infection progresses or the toxin concentration increases, the role of Hla shifts to inducing inflammatory cell death, thereby reducing the number of immune cells and amplifying tissue damage. Firstly, Hla can activate the RIPK1 (serine-threonine kinase 1) - RIPK3 (serine-threonine kinase 3) - MLKL (human mixed lineage kinase domain-like protein) signaling pathway, triggering necrotic apoptosis (25). Secondly, the intracellular K^+^ efflux mediated by Hla provides an activation signal for the NLRP3 inflammasome, and after NLRP3 activation, it can promote the activation of caspase-1 and the subsequent release of pro-inflammatory factors such as IL-1β and IL-18 (26–28).

In addition to the lethal effects, Hla can also “paralyze” the bactericidal ability of macrophages through non-lethal pathways. Hla can synergistically inhibit phagocytic efficiency by interacting with the bactericidal factor LukAB (29), and block the acidification process of bacterial phagosomes (30). At the same time, Hla disrupts the co-localization of mitochondria and phagosomes, thereby destroying the mitochondrial-dependent reactive oxygen species (ROS) antibacterial defense system (31). Therefore, Hla not only reduces immune cells through “killing” but also weakens their effect functions through “deactivation”, thereby promoting immune disintegration at multiple levels.

The RTX toxin expressed by *Filifactor alocis* also has the effects of inducing inflammatory cell death and immunosuppression. However, its concentration-dependent effect is different from that of Hla. The research results show that FtxA acts on THP-1 macrophage-like cells in a non-cleaving manner, and by systematically down-regulating the NF-κB, TNF-α and TLR signaling pathways, it inhibits the expression of key inflammatory cytokines such as IL-1β and CXCL8 (32). Meanwhile, FtxA can also actively inhibit exogenous apoptotic signals and the apoptotic pathways mediated by p53, prolonging the survival of host immune cells, thereby creating an immunosuppressive microenvironment conducive to the persistent colonization of this obligate anaerobic bacterium. This immunoregulatory strategy contrasts with the hla concentration-dependent bidirectional effect, revealing the host adaptation mechanisms differentiated at different infection sites.

At the level of adaptive immunity, T lymphocytes exhibit a high sensitivity to Hla, but there are significant differences in susceptibility among different subgroups. Existing studies have shown that Hla tends to preferentially kill Th1 cells that mediate protective immunity, while its killing of Th17 cells is relatively weak (possibly related to the specific tolerance of Ca^2+^ signal transduction in Th17 cells) (33–35). This differentiated killing mechanism can promote the immune response to shift towards the Th17 type, although this pathway can recruit a certain number of neutrophils, it is often insufficient to achieve complete clearance and instead may create conditions for the maintenance of chronic infections (36, 37).

What is more serious is that Hla can also induce the death of antigen-specific T cells, thereby blocking the establishment of immune memory. In the skin infection model, the T cell exhaustion mediated by Hla makes it difficult for the host to form effective long-lasting immune memory, which provides an important explanation for the common recurrence and lack of lasting immunity in clinical *S. aureus* infections (38). Moreover, in the related research on skin T-cell lymphoma (CTCL), it was also observed that Hla can specifically eliminate CD8+ cytotoxic T cells, thereby simultaneously promoting immune escape at both the tumor and pathogen levels (38).

In summary, Hla employs a phased strategy of “early promotion of survival - later promotion of inflammatory death”, on the one hand, shaping intracellular ecological niches conducive to bacterial proliferation within innate immune cells; on the other hand, inducing necroptosis and pyroptosis after toxin accumulation and triggering an inflammatory cascade reaction. At the subcellular level, the Hla secreted by *Staphylococcus aureus* within the cell can escape from the phagosome and further target the lysosomal membrane. Through its heptameric RIM region’s Loop 68-75, it forms a high-affinity binding with the carbohydrate recognition domain of the lysosomal damage sentinel protein LGALS3, competitively inhibiting the recruitment of the ESCRT repair protein PDCD6IP/ALIX by LGALS3, thereby blocking the repair of the damaged lysosomal membrane. This leads to the loss of lysosomal acidification (the pH increases from 4.56 to 5.48) and the paralysis of degradation function, preventing the autophagosome enclosing the bacteria from completing its clearance. This provides a crucial refuge for the intracellular persistence and recurrence of the pathogen (39).At the same time, Hla’s multi-target intervention on phagosome maturation, the ROS antibacterial system, and T cell subsets and immune memory ultimately leads to the systematic disintegration of the host’s immune defense network. It is worth emphasizing that this immune disintegration is not an isolated event. Intense inflammation amplification and cell membrane damage will further spill over to continuously attack the local tissue microenvironment and barrier structure.

#### 2.1.2 The “double-edged sword” effect of the acidic sphingomyelinase-neuroceramide system

In addition to directly killing immune cells, the core harm of Hla to non-immune host cells (such as endothelial cells) lies in disrupting the integrity of the tissue barrier. During this process, the ASM-neuroceramide system plays a crucial central role. In the case of lower toxin load or early stages of action, this system is more like an emergency repair mechanism, helping cells remove pores and maintain membrane integrity. When the concentration of Hla increases or persists, this “repair function” transforms into a “destructive function”, ultimately leading to the disintegration of intercellular connections and uncontrolled barrier permeability.

The direct substrate of ASM is sphingomyelin (SM) in the outer leaflet of the cell membrane. Therefore, the abundance and distribution of SM may affect the efficiency of Hla’s action on the membrane. Consistent with this, studies have shown that cells lacking sphingomyelin synthase 1 (SGMS1) are resistant to the virulence of Hla (40). This indicates that the barrier induced by Hla is not merely a pore effect but a result of the combined action with the host’s membrane lipid metabolism. Research has shown that SM in the PM is essential for the formation of Hla heptamers, but the presence or absence of SM does not affect the binding of Hla to the PM (41). However, after eliminating SM with Hlb as a neutral sphingomyelin, a small portion of polymers could still be observed (14). This might be because choline-containing lipids (such as PC) could replace SM to a certain extent and mediate the formation of Hla heptamers. Another possible explanation is that Hla monomers could spontaneously assemble at a low rate without any lipids (41).

In the early stage of Hla attack or under low concentration conditions, transmembrane pores can cause ion exchange disorder. Among them, the inward flow of Ca^2+^ is often the key “triggering signal” for the repair response. An increase in Ca^2+^ can drive the rapid recruitment and exocytosis of lysosome-associated protein 1 (LAMP1) rich in ASM, causing the externalization of ASM to the outer side of the cell membrane (42). Subsequently, the exocytosed ASM hydrolyzes sphingomyelin on the outer leaflet of the plasma membrane to generate ceramides, promoting Ca^2+^-dependent endocytosis of the pore (43). The endocytosis of the Hla toxic pore can re-close the damaged cytoplasmic membrane of the Hla-infected cells (44, 45). Therefore, at this stage, the main role of ASM-neuroceramide is more closely related to the protective self-rescue mechanism of “clearing the pore - maintaining membrane integrity”, which can help the cells survive the acute membrane damage to a certain extent.

When the concentration of Hla increases or the toxin effect persists, the activation of ASM will severely degrade the stability of intercellular connections. Previous studies have shown that ADAM10 is a key factor in Hla-induced cytotoxicity, and its activation is related to the degradation of tight junction proteins (such as E-cadherin) (46, 47). At the same time, ceramides can mediate the release of tissue proteases B and D into the cytoplasm, further degrading tight junction-related proteins and accelerating the disintegration of the connection structure (48, 49). The direct consequence of these changes at the tissue level is a significant increase in the permeability of endothelial and epithelial cells, which is closely related to the lethal pulmonary edema caused by *S. aureus* infection and the dissemination of bacteria to the bloodstream (50).

In summary, the ASM-neuraminic acid system holds a “central hub” position in Hla-mediated barrier damage. At low toxin loads, it achieves rapid closure of the plasma membrane breach and maintenance of cell survival through Ca^2+^-triggered lysosomal exocytosis and membrane lipid remodeling. However, under high concentration or continuous exposure conditions, this system becomes overactivated, resulting in pathological accumulation of neuraminic acid and ultimately pushing “local repair” to “structural failure”, manifested as a sharp increase in epithelial/endothelial barrier permeability and severe tissue damage. The direct pathological consequences of this barrier disruption in the body were verified in a skin infection model: Hla induces apoptosis of dermal vascular endothelial cells by cleaving the VE-cadherin of vascular endothelial cells in the early stage of infection (6 hours), causing local vascular damage; the loss of vascular integrity further leads to tissue ischemia and hypoxia, and triggers extensive epidermal necrosis (dermal necrosis) within 24 hours. Using Hla neutralizing antibodies or ADAM10 inhibitors can significantly alleviate early vascular damage, relieve tissue hypoxia, and prevent the occurrence of dermal necrosis. This confirms at the therapeutic level that the disruption of the vascular endothelial barrier is the core driving skin tissue necrosis by Hla (51).

#### 2.1.3 Regulation of host defense: mechanisms of inflammation clearance and resolution

During the co-evolution of the host and *S. aureus*, the inflammatory response induced by Hla exhibits a significant “double-edged sword” effect. On the one hand, the inflammatory signals activating the “low-level” state are insufficient to eliminate the bacteria (52); on the other hand, excessive or uncontrolled inflammation will amplify tissue damage (53). The host has evolved regulatory mechanisms capable of sensing Hla-related signals, so as to initiate the inflammatory resolution and tissue repair programs after effectively eliminating the pathogen, thereby avoiding excessive defense reactions that cause damage to the host.

The initial defense against the infection site relies on the rapid recruitment of neutrophils, and Hla is considered one of the important chemotactic signals that induce this process (54). Neutrophils usually enter the apoptotic mechanism after completing the bactericidal task. If neutrophils cannot be cleared in time, they will release a large amount of toxic particles and proteases, further aggravating local tissue damage and forming “excessive inflammation” (55). To address this risk, the host can establish a balance through scavenger receptors CD36 on the surface of macrophages. Studies have shown that CD36 can specifically recognize and engulf the apoptotic neutrophils affected by Hla, thereby limiting the expansion of the necrotic focus and significantly reducing tissue damage (53). In summary, the host does not passively endure the inflammation driven by toxins but inhibits the destructive overflow by improving the efficiency of inflammation clearance.

**Figure 1:**
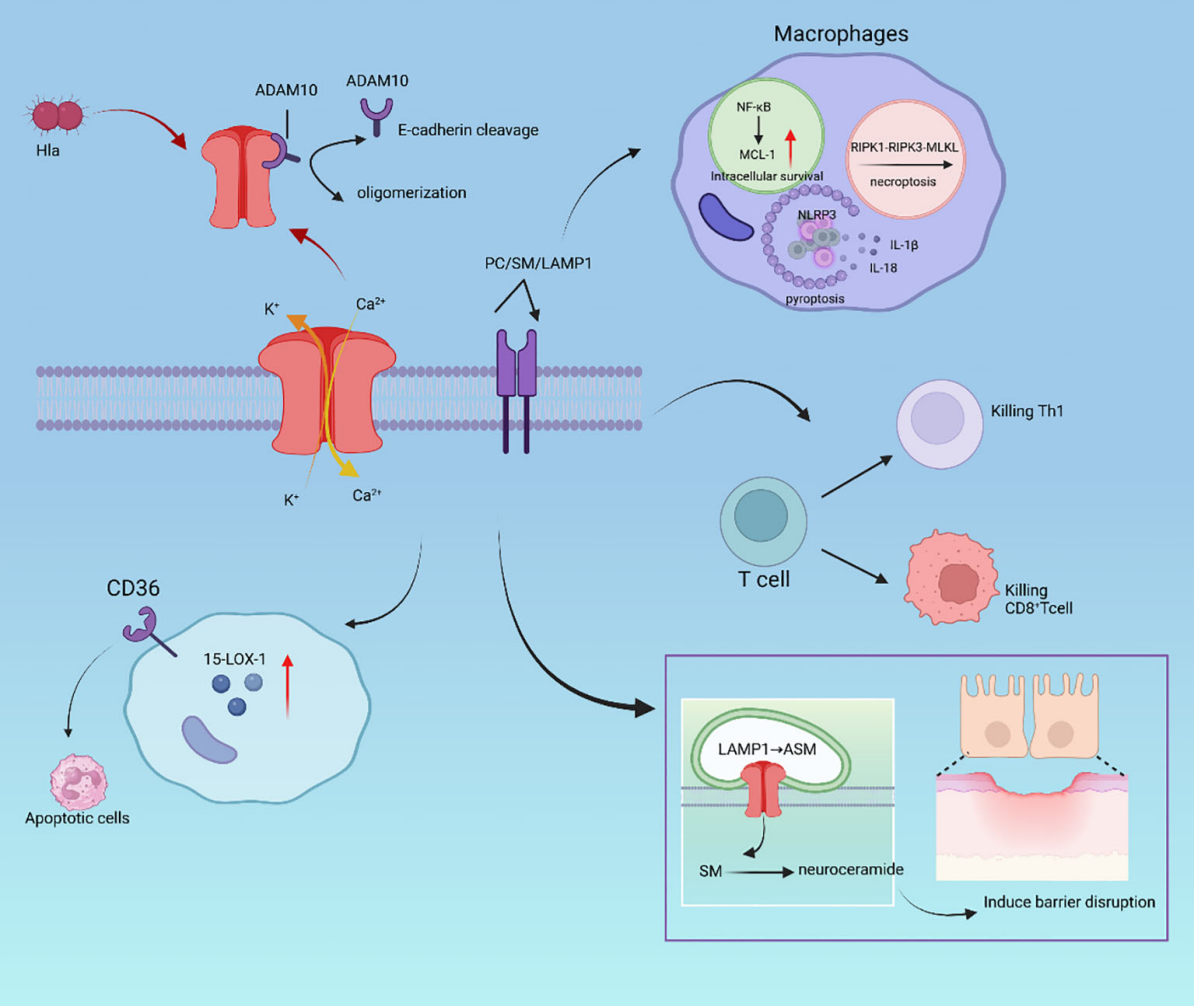
Hla toxicity effect. In macrophages, Hla undergoes E-cadherin cleavage and oligomerization mediated by ADAM10, regulating PC/SM/LAMP1; it also causes K^+^ and Ca^2+^ ion flow; Hla can activate NF-κB and MCL-1 to achieve intracellular survival, activate the NLRP3 inflammasome to produce IL-1β/IL-18, inducing pyroptosis of cells, and trigger necroptosis through the RIPK1-RIPK3-MLKL pathway; it kills Th1 and CD8^+^ T cells; LAMP1 activates acid sphingomyelinase (ASM) to generate sphingomyelin (SM) and ceramides, inducing barrier disruption; by upregulating CD36, it promotes the clearance of apoptotic cells.

Further studies have shown that the host may even use Hla as a signal to initiate repair, pushing the inflammation from the clearance phase to the repair phase in the later stage of infection. Jordan et al. reported that human macrophages exposed to Hla can upregulate the expression of 15-lipoxygenase-1 (15-LOX-1), which is a key factor in synthesizing pro-degradative mediators (SPMs). Unlike pro-inflammatory lipid mediators (LMs) (such as prostaglandin PG and leukotrienes LTs), SPMs can promote inflammation resolution and tissue regeneration and restore the host to homeostasis (56–58). In summary, Hla can not only drive inflammation and tissue damage but also be incorporated by the host into a negative feedback regulatory network, achieving orderly shutdown of inflammation and tissue repair through “clearing apoptotic cells and generating SPMs”. The Hla toxic effect is shown in **Figure 1**.

### 2.2 The phased functional characteristics of Hla during the infection process

Based on the different clinical stages of *Staphylococcus aureus* infection, this article divides the functional evolution of Hla into three consecutive but possibly overlapping periods: the early stage (local infection and initial colonization), the progression stage (infection spread and immune imbalance), and the late stage (systemic dissemination and barrier disruption). The early stage corresponds to the local soft tissue infection or initial pneumonia stage in clinical terms, at which time the bacteria are confined to the primary site, and Hla is expressed at a low level, mainly participating in immune regulation and promoting survival; the progression stage corresponds to the spread of infection to deeper tissues or adjacent areas, accompanied by local necrosis and uncontrolled inflammation, and Hla accumulates to a level that can induce the massive death of immune cells and excessive activation of inflammasomes; the late stage corresponds to bacteremia, sepsis, or multiple organ dysfunction, where Hla remains highly expressed, directly destroying the epithelial/endothelial barrier, promoting the systemic dissemination of bacteria and aggravating organ damage. It should be noted that the boundaries between these stages are not absolute but depend on the host’s immune status, bacterial load, and the dynamic interaction of the local microenvironment. The main functional characteristics of Hla in each stage are described below.

#### 2.2.1 The functional characteristics of Hla during the early stage of infection

In the early stage of infection, the production level of Hla is relatively low, and its function is usually confined to the local microenvironment. At this stage, Hla does not mainly manifest as large-scale cell killing but rather participates more in regulating the host’s immune response. The low-level toxin signals can affect the survival status of innate immune cells, by inhibiting apoptotic pathways, providing conditions for *S. aureus* to establish a relatively stable ecological niche within cells or in local tissues (24). At the same time, the Ca^2+^ influx induced by Hla is more likely to trigger membrane repair-related reactions, enabling the target cells to survive after experiencing acute membrane damage (43). At this time, the intensity of the generated inflammatory signals is limited, which can disturb the host defense system but is not sufficient to trigger uncontrollable tissue damage. Overall, the functional characteristics of Hla in the early stage of infection are more inclined towards promoting survival and immune regulation, which is conducive to the bacteria completing the initial colonization and escaping immune clearance.

#### 2.2.2 Immune imbalance mediated by Hla during the progression of infection

As the infection persists, the local Hla levels gradually increase, and the impact of this on the host’s immune system begins to undergo a qualitative change. At this stage, the accumulation of toxins leads to an increase in the frequency of transmembrane pore formation, and the outward flow of K^+^ and the activation of inflammasomes become more significant. Pro-inflammatory cell death gradually becomes the dominant outcome (26–28). The loss of a large number of immune cells and the concentrated release of inflammatory factors amplify the local inflammatory response and weaken the effective immune defense ability.

Furthermore, during the progression of the infection, Hla can further exacerbate the imbalance of the immune system by interfering with phagocytic function, reactive oxygen species production, and antigen-specific T-cell responses (29–31). The impaired protective immune response and persistent low-level inflammation allow the infection site to expand. The key feature of this stage is that Hla no longer mainly plays a regulatory role but instead amplifies inflammation and weakens immune effects, creating conditions for the continuous proliferation of bacteria.

#### 2.2.3 Hla mediates the destruction of the tissue barrier during the later stage of infection

In the later stage of infection, the continuous action of Hla poses a significant challenge to the integrity of the epithelial and endothelial barriers. The high-level exposure to toxins leads to the long-term existence of Ca^2+^ influx and ion homeostasis disruption, the gradual depletion of the cell membrane repair system, impaired mitochondrial and lysosomal functions, and the accumulation of pro-inflammatory death or severe dysfunction in structural cells.

When such damage occurs in the barrier tissues, its impact rapidly spreads from the single-cell level to the tissue level. Cell death and unstable connection structures jointly lead to an increase in permeability, which can manifest as edema formation and functional impairment in organs such as the lungs, while also increasing the risk of bacteria spreading to deeper tissues or the bloodstream. As a result, the function of Hla in the later stage of infection is more concentrated on barrier disruption and promotion of transmission, but it also significantly increases the risk of the host developing severe diseases.

It is worth noting that the expression level of Hla varies significantly among different clinical isolates of *Staphylococcus aureus*. This heterogeneity directly affects the pathogenic potential and clinical manifestations of the strains. Zhang et al. reported a class of clinical isolates of *Staphylococcus aureus* (SIHP) that exhibited an “incomplete hemolysis phenotype”. Compared with the strains with a complete hemolysis phenotype (SCHP), the transcriptional level of the hla gene in SIHP was significantly suppressed by 50 times, the expression of α-hemolysin protein was reduced by approximately 40 times, while the expression of β-hemolysin (hlb) was instead upregulated by 7.7 times. All SIHP strains belonged to MRSA, carried the mecA and tst virulence genes, had significantly enhanced survival ability in macrophages, and could induce higher levels of IL-2, IL-6, and IL-17A secretion (59). The expression of TSST-1 and Hla seems to be in a mutually inhibitory relationship, suggesting that it may promote cell survival. In closely related species, the expression levels of Hla in Staphylococcus argenteus and Staphylococcus schweitzeri are 12–15 times higher than that in *Staphylococcus aureus*. The expression levels of their regulatory factors sarA, saeR, and RNAIII in the agr system also increase significantly (the increase in RNAIII can reach 8, 000-10, 000 times), resulting in a strong necrotic cytotoxicity of the culture supernatant towards human epithelial cells. This effect can be completely neutralized by anti-Hla antibodies (60). These findings indicate that the expression of Hla is precisely controlled by the genetic background of the strain and the regulatory network. The level of Hla expression is directly related to the hemolytic phenotype, intracellular survival ability, and cytotoxicity. The heterogeneity of Hla expression in clinical strains may lead to the diversification of infection manifestations - ranging from acute necrotic damage to chronic diseases characterized by immune evasion and intracellular persistent infection.

## 3 β-hemolysin

β-hemolysin (Hlb) is a secreted protein composed of approximately 330 amino acids and with a molecular weight of about 39 KDa. It can exert toxic effects on various immune cells such as polymorphonuclear leukocytes, monocytes, and T lymphocytes (61, 62). Unlike the pore-forming effect dependent on Hla and the triggering of ion homeostasis disruption within a short period of time that leads to acute cell death, the distinctive feature of Hlb is that it acts as a neutral sphingomyelinase (SMase), specifically degrading sphingomyelin into ceramides and phosphorylcholine, which is crucial for damaging the target cell membrane (63, 64).

This mode of action determines that the “functional positioning” of Hlb during the infection process is often different from that of acute destructive toxins. Hlb does not necessarily rely on strong hemolysis or direct lethality as its main output, but rather more often manifests as the regulation of colonization niches, the vascular endothelium-platelet axis, the stability of biofilms, and the rhythm of inflammatory responses. Therefore, although its acute cell lysis effect is relatively limited, Hlb is likely to have an irreplaceable contribution in determining the persistence, protracted nature, and course of the disease of the infection.

### 3.1 The toxic effect of Hlb

#### 3.1.1 Establishment of local ecological niche: promote bacterial colonization and the stability of biofilms

As the first step, Hlb assists in the adhesion and persistent presence of bacteria at the infection site. Most clinical isolates of *Staphylococcus aureus* do not consistently express Hlb. The main reason for this is that the φSa3int phage family is widely integrated into the structural gene hlb of β-hemolysin, rendering it inactive. However, this “inactivation” is not permanent. The phage can respond to environmental changes and be removed. During the infection process, oxidative stress and the formation of bacterial biofilms can induce the removal of the phage (bacterial biofilm refers to the situation where bacteria adhere to the contact surface and form a large number of bacterial aggregations by secreting polysaccharides, fibrin, lipoproteins, etc., forming a membrane-like substance around themselves) (65). The removal of the phage can lead to a time-dependent recovery of Hlb production. This “inducible recovery” characteristic makes Hlb more like a functional module that is activated by bacteria on demand.

In the *S. aureus* MW2 strain, the colonization ability of the bacteria on the skin of mouse ears significantly increased after the phage φSa3int was removed, accompanied by damage to keratinocytes (66). This phenomenon suggests that the restoration of Hlb expression may change the local barrier surface environment, thereby facilitating bacterial adhesion and residence. Additionally, in the human nasal mucosa colonization model, the strain carrying the φSa3 phage only colonized for 4 days, while the strain without φSa3 could colonize for up to 14 days (67). This provides further support for the role of Hlb in maintaining mucosal colonization.

At the biofilm level, the DNA biopolymerization enzyme activity of Hlb has been demonstrated to promote the formation of covalent nuclear protein complexes, thereby enhancing the structural stability of the biological membrane matrix (68). In the rabbit model of infectious endocarditis, an increase in Hlb levels can increase the volume of the biofilm on the surface of the heart valve and the size of the plaque (69). It is noteworthy that this effect is not necessarily accompanied by a significant increase in the bacterial proliferation rate within the biofilm, suggesting that Hlb is more likely to enhance the persistent infection ability by “strengthening the structure” and “improving tolerance”, rather than simply promoting proliferation.

#### 3.1.2 Reconfiguration of the deep tissue microenvironment: the endothelial-platelet axis and anti-angiogenesis

Once the bacteria break through the local barrier, the role of Hlb shifts to remodeling the vascular and stromal environment. This process is achieved through two mutually reinforcing mechanisms: a)During the process of invasive infection, Hlb can regulate the functions of endothelial cells and platelets to create a microenvironment more conducive to the survival, concealment and expansion of bacteria. In the infection-induced endocarditis model, Hlb can inhibit the expression of CD40 on the surface of endothelial cells and vascular cell adhesion molecule 1 (VCAM-1) and reduce the production of chemokine IL-8 (70). Such changes often indicate that in the early stage of infection, there is a “lack of sufficient inflammatory signals” locally: delaying the activation of the host immune system, allowing bacteria to have a larger time window to complete adhesion and colonization. Meanwhile, Hlb can also induce platelet aggregation through integrin-dependent mechanisms, thereby promoting coagulation and the formation of plaques (70). Unlike Hla, which promotes diffusion through the acute destruction of the barrier, Hlb is more like “rearranging the host surface” to construct a relatively stable platform, allowing the bacteria to exist and expand there for a longer period of time.

##### 3.1.2.1 Anti-angiogenic effect and impaired tissue repair

The interference of Hlb with the angiogenesis and re-endothelialization processes is one of the important mechanisms by which it promotes invasive infections. The dynamic balance of tissue repair depends on angiogenesis, which is determined by both pro-angiogenic factors (including VEGF, HGF, serpin E1, EGF, bFGF, endothelin-1, and PDGF) and inhibitory factors (including thrombomodulin and endostatin) (71, 72). Hlb can reduce the production of endothelin-1 and thrombosis response protein-1 in endothelial cells. Endothelin-1 is an effective mitogen for endothelial cells, which can stimulate migration and contribute to the integrity of endothelial cells, which is particularly important in newly formed blood vessels (73). Thrombomodulin-1 is a non-structural extracellular matrix protein that acts as an effective endogenous inhibitor of cell adhesion, migration, and proliferation. Its main function is to counteract the effects of angiogenic stimulation and effectively shut down the angiogenesis switch (74). Additionally, Hlb also reduces the production of TIMP-1, TIMP-4, and IGFBP-3. It is known that TIMP can control MMP activity to maintain extracellular matrix homeostasis and promote germination, vascular stability, and vascular regression (75). However, whether IGFBP-3 induces or inhibits cell migration depends on the cell type. Therefore, Hlb may lead to an imbalance in protein production, thereby accumulating to disrupt angiogenesis in immortalized human aortic endothelial cells (iHAEC) (76).

Under the condition of wound healing, Hlb can reduce the expression of the pro-angiogenic molecule MMP-8 and simultaneously increase the production of the anti-angiogenic factor endostatin (76). The combination of these target changes will lead to the obstruction of vascular regeneration, causing the affected tissue to remain in a state of incomplete repair for a long time. In the context of infection, the impact of blocked vascular repair is not limited to “slower wound healing”. More importantly, insufficient angiogenesis and re-endothelialization may reduce the efficiency of immune cells and drugs entering the lesion site, making the infected area more likely to evolve into a persistent and protracted local ecological niche, thereby indirectly promoting the progression of invasion.

These two mechanisms work together to transform the local tissue from an “hostile environment” with immune capabilities into an “allowable ecological niche” that supports the long-term persistence of the bacteria.

#### 3.1.3 Biphasic immune regulation: from immune escape to chronic inflammation

The effect of Hlb on the inflammatory response is not simply promoting or inhibiting in a single direction, but rather it is related to the differences in the infection stage, toxin levels, and tissue background. In the early stage of infectious endocarditis, Hlb can interfere with the EGFR-related pathways, inhibit the phosphorylation of ERK1/2, thereby reducing the production of IL-8 by endothelial cells and delaying the recruitment of immune cells (70). This “inhibitory effect on chemotactic signals” is consistent with its function of promoting colonization and helping bacteria overcome the initial immune pressure.

As the infection progresses and Hlb expression recovers and persists, the sphingomyelinase activity of Hlb may again promote inflammation amplification in certain tissues. For example, in an inflammatory skin disease model, Hlb activates EGFR through its sphingomyelinase activity to induce skin inflammation (77). During this process, Hlb causes phosphatidylserine (PS) to be exposed from the inner cell membrane vesicles to the outer cell membrane vesicles. The exposure of PS is crucial for the shedding enzyme activity of ADAM17. After ADAM17 is activated, it leads to an increase in the release of ligands that detach from EGFR, and subsequently, the released soluble ligands bind to EGFR and activate the EGFR signaling pathway (77). Furthermore, Hlb can upregulate the production of IFN-γ by CD56^bright^ NK cells in human peripheral blood mononuclear cells (PBMCs) (78). This process is associated with calcium influx and ERK1/2 signaling and is dependent on the sphingomyelinase activity of Hlb. When calcium influx is blocked or specific inhibition of ERK1/2 phosphorylation is applied, the IFN-γ induced by Hlb is reduced (78). Considering that IFN-γ is associated with various chronic inflammatory diseases (79–81), these findings suggest that Hlb may participate in the formation and maintenance of chronic inflammatory states by regulating the inflammatory network in a long-term and low-level manner.

**Figure 2:**
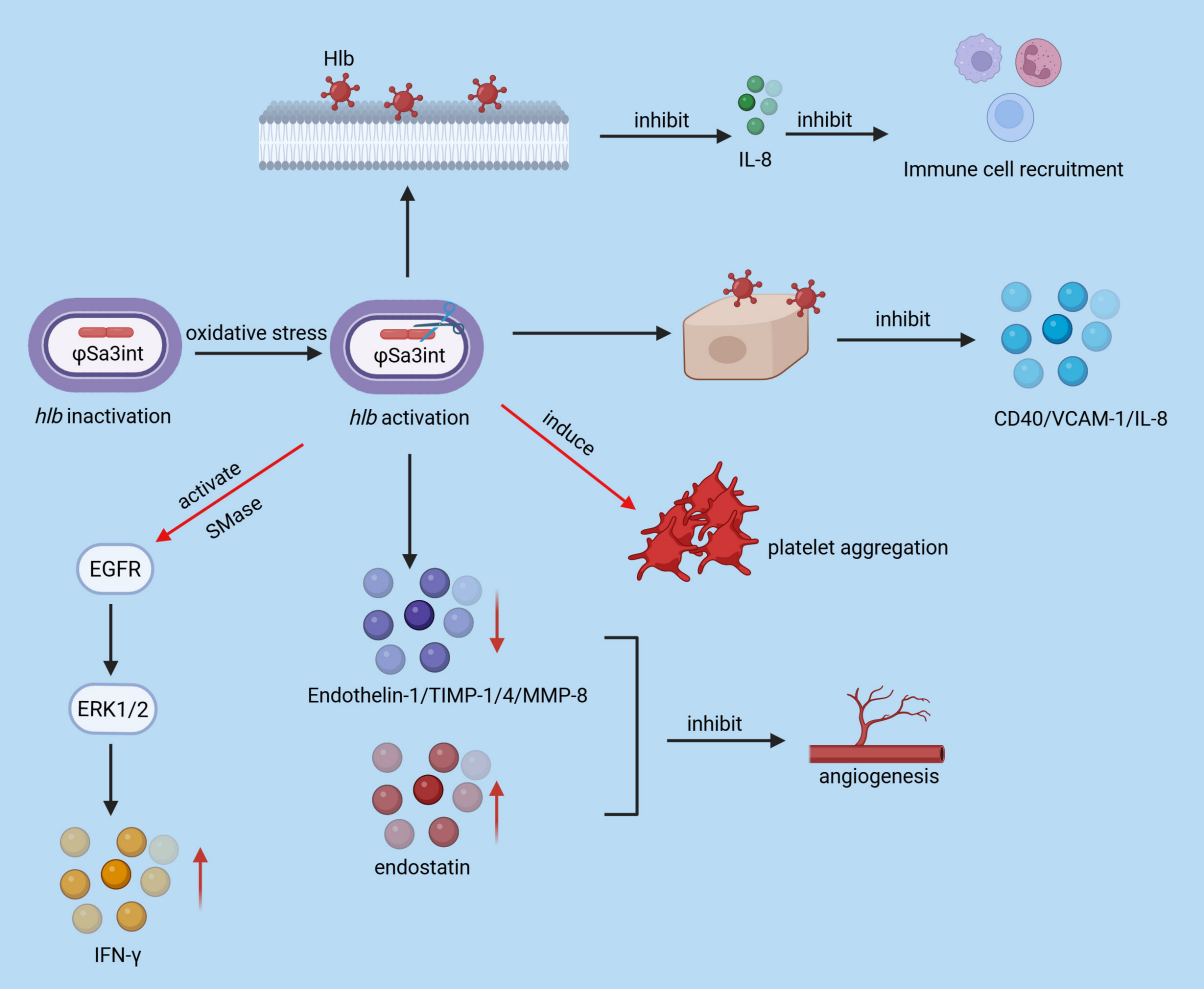
Hlb toxicity reaction. Hlb can inhibit the production of IL-8 and the recruitment of immune cells; oxidative stress leads to the inactivation of Hlb by inducing the φSa3int bacteriophage; meanwhile, Hlb can activate SMase, EGFR, ERK1/2 and IFN-γ, induce platelet aggregation, and regulate the expression of Endothelin-1, TIMP-1/4, MMP-8 and endostatin, thereby affecting angiogenesis.

Therefore, the role of HLB is dual: in the early stage, it inhibits the host’s immune control to help evade, while in the later stage, it promotes the development of a persistent inflammatory state. The threshold for transitioning from anti-inflammatory to pro-inflammatory effects and the tissue-specific determinants of this process require further research using *in vivo* models. The toxic reaction of HLb is shown in **Figure 2**.

According to the existing literature, the current understanding of the expression of β-hemolysin (Hlb) in clinical isolates of *Staphylococcus aureus* is as follows:

The epidemiological survey indicates that the hlb gene has a relatively high carrying frequency in clinical isolates. For instance, Lin (82) isolated 33 strains of *Staphylococcus aureus* from sterile body fluid specimens, and the positive rate of the hlb gene reached 96.97% (32/33), with no significant difference between MRSA and MSSA strains. Orellana (83) isolated 14 strains of bacteria from the nasal cavities of medical staff, and the positive rate of the hlb gene was 57% (8/14), while the positive rate of the methicillin-resistant *Staphylococcus aureus* (MRSA) isolates was 50% (1/2). However, gene carrying does not equate to protein expression. The expression of hlb is regulated by prophage insertion: when the hlb gene is inserted with phages such as φSa3mw, the production of β-hemolysin is interrupted. Liu (84) study revealed that some clinical strains (such as NRS049) carry the hlb gene but, due to the absence of prophage insertion, can normally express β-hemolysin and subsequently inhibit the hemolytic phenotype of α-hemolysin. This suggests that there may be differences between the hlb genotype and the functional phenotype of β-hemolysin, and the assessment of hlb expression in clinical isolates requires a combination of genetic testing and phenotypic verification. In general, the hlb gene is widely present in clinical isolates, but its actual expression is influenced by the integration status of prophages and the genetic background. Its biological significance in different infection types and colonization states still requires further research.

### 3.2 The phased functional characteristics of Hlb during the infection process

#### 3.2.1 Early stage of infection: limited expression of Hlb and the initial colonization window

In most clinical isolates, the Hla gene is integrated and inactivated by the φSa3int phage, resulting in a state of low or absent expression of Hlb during the early stages of infection (65). This expression-restricted state may reduce the triggering of additional inflammation in the early stage of infection, thereby helping the bacteria complete the initial adhesion and colonization during the window period when the host immune system is highly alert. At this time, the bacteria often rely more on other surface proteins and immune evasion factors to establish an ecological niche, and Hlb does not need to be activated immediately. However, the lysis of HLB-transformed bacteriophages is a dynamic and reversible process - environmental stresses such as chronic infection, oxidative stress, and antibiotic exposure can all induce lysis of the bacteriophages and restore the expression of functional HLB (85). Enable the bacteria to acquire sphingomyelinase activity during the middle and later stages of infection, thereby degrading the sphingomyelin in the red blood cell membrane and impairing the oxygen transport function (86). This further aggravates tissue hypoxia and disease progression during the deep infection or chronic stage.

#### 3.2.2 Progression stage of infection: stress-induced expression and colonization/biofilm enhancement

As the bacterial load increases and the local microenvironment deteriorates (with intensified nutrient competition, biofilm formation, etc.), phage excision can be induced, allowing Hlb expression to recover in a time-dependent manner (65). This transformation often indicates a shift in the infection strategy from “trying not to draw attention” to “beginning to modify the host surface and the local microenvironment”.

At this stage, HLb delays the growth of immune cells by exerting its functions in colonization enhancement and biofilm reinforcement, as well as by inhibiting the output of endothelial cell chemotactic factors. This reduces the conversion time of the biofilm from “initialization” to “fixation”. Moreover, the anti-angiogenic effect of HLb begins to damage the local tissues, helping the bacteria survive under high immune pressure. The specific length and intensity of this period and its dependence on toxin load still need to be defined through *in vivo* experiments.

#### 3.2.3 Late stage of infection: continuous membrane lipid remodeling and progression of invasion/chronicization

When the infection reaches the later stage, the bacteria have established a stable ecological niche within the tissues and may spread to deeper tissues or the bloodstream. At this point, the continuous action of Hlb is more likely to manifest as long-term disturbances to the barrier, vascular repair, and inflammatory networks. At this point, the function gradually shifts from suppressing the immune system to promoting chronic inflammation.

Specifically, Hlb’she continuous hydrolysis of sphingomyelin and the accumulation of ceramides may lead to a slow weakening of the barrier, providing “structural gaps” for the bacteria to cross the endothelium. Secondly, the anti-angiogenic effect of Hlb will delay re-endothelialization and tissue repair, making it more difficult to effectively seal the infection site, and also reducing the efficiency of immune cells and drugs to enter the deep lesion area (74). At the same time, Hlb later shifts to an inflammatory state. The organization can maintain low-level but persistent inflammatory signals (such as amplification of EGFR-related pathways and upregulation of IFN-γ), and this inflammatory intensity may not be sufficient to eliminate the bacteria, but it may continuously damage the surrounding tissues, thereby promoting chronicity and recurrent attacks (77, 78).

## 4 Antitoxin therapy

At present, it cannot be completely replaced, but it has great potential to become an important auxiliary or alternative solution. Due to the high resistance of MRSA and VRSA to traditional antibiotics (such as methicillin and vancomycin), direct bactericidal treatment is facing a bottleneck. The anti-virulence strategy targets the pathogenic factors of bacteria (such as hemolysins, biofilms, etc.) rather than the essential genes for bacterial survival. In theory, it is less likely to induce drug resistance and can work synergistically with existing antibiotics. However, its limitation lies in the inability to eliminate the bacteria themselves, and in severe infections, it still needs to be combined with bactericidal drugs. The following text will elaborate on the differentiated anti-toxic mechanisms targeting two key hemolysins, providing a theoretical basis for evaluating the feasibility of this strategy.

In the research on antitoxin treatment for *Staphylococcus aureus* infections, there are fundamental differences in the mechanism of action of different hemolysins. The target selection can be divided into two major categories: directly inhibiting the biological activity of the hemolysins themselves (such as blocking pore formation, enzymatic activity or receptor binding) and interfering with the host cell’s response to the toxins or the downstream signaling pathways activated by the toxins (such as blocking calcium influx, inflammasome activation or specific receptor signaling).

The strategy for anti-toxin against α-hemolysin is based on blocking its pore-forming cascade reaction, which belongs to a direct targeting strategy towards the toxin’s activity. α-hemolysin is a pore-forming toxin. Its pathogenic process involves binding to the receptor membrane of host cells, achieving oligomerization into a heptameric pre-pore, and ultimately forming a functional transmembrane channel. Current research mainly focuses on the following aspects: On the one hand, directly binding to the monomers of the toxin to prevent their isomerization. The quinaginyl small molecule inhibitors reported by Shekhar (87). can directly interact with α-chitinase, preventing the formation of functional heptamer channels, thereby effectively inhibiting the cell damage mediated by the toxin both *in vivo* and *in vitro*. Additionally, they block the connection between the toxin and the host receptor. Take Sakara et al. as an example. Smith stated in the report that multiple anti-α-lactamase monoclonal antibodies (such as MEDI4893/suvratoxumab) can neutralize the toxin, preventing its binding to ADAM10 or its self-separation (88). Thirdly, through the use of vaccines to stimulate high-titer neutralizing antibodies, the HlaHRE vaccine developed by Tomaszewski, by introducing specific mutations, not only has the potential to induce a stronger neutralizing antibody response than traditional toxoids but also can protect the helper response of T follicular cells in the body, thereby effectively preventing infection in newborn mouse models (89). Additionally, some new compounds can inhibit the expression of α-antitoxin at its source, For instance, Hao (90) discovered that iclaprim inhibits the transcription and expression of HLA gene proteins at sub-inhibitory concentrations; Wang (91) found that the natural product Pikeatono can directly bind to multiple sites, such as the edge domain and the n-terminus of α-hemolysin, among others. This affects the binding and assembly of the membrane, ultimately inhibiting the hemolytic activity.

The mechanism of β-hemolysin is completely different from that of α-hemolysin. The core lies in inhibiting its sphingomyelinase activity and the downstream signaling pathways it triggers. β-hemolysin is a toxin that relies on enzymatic activity. The classical pathogenic mechanism is to hydrolyze cell membrane sphingomyelin, leading to cell lysis, but this process requires alternating use of cold and heat under non-physiological conditions. The latest research by Li (86). emphasizes a new pathogenic mechanism unrelated to hemolysis at physiological temperatures: β-hemolysin destroys the red blood cell membrane’s sphingomyelin through its sphingomyelinase activity, causing the disruption of lipid raft structures, and subsequently activating the N-methyl-D-aspartate (NMDAR) receptor channels, resulting in a large influx of calcium ions. The intracellular calcium overload triggers cytoplasmic alkalization, ultimately interfering with the oxygen transport and release of red blood cells. Therefore, the anti-toxin strategy targeting β-hemolysin should aim to inhibit its sphingomyelinase activity or block the NMDAR-calcium signal (for example, using NMDAR inhibitors such as mk-801). This viewpoint proposes a new therapeutic direction to improve tissue hypoxia caused by *Staphylococcus aureus* infection.

In summary, the mechanism of α-hemolysin antitoxin is to block its physical pore-forming process, while the mechanism of β-hemolysin is to inhibit its enzymatic activity and signal transmission. This provides a theoretical basis for the development of differentiated anti-toxin resistance.

## 5 Conclusions

From the perspective of molecular mechanisms, the pathways through which these two drugs cause cell damage are completely different. α-hemolysin (Hla): Its pathogenic process begins with the specific recognition of host cell membrane receptors. Hla first enriches in lipid raft microdomains through phosphatidylcholine (PC), providing a platform for the oligomerization of the toxin. Subsequently, its core protein receptor ADAM10 is recruited and binds. This binding event not only serves as an “anchor point” for the toxin but also directly triggers the abnormal activity of ADAM10 metalloproteinase. The activation of ADAM10 leads to the “splitting” of cell-binding proteins (such as E-cadherin), disrupting the integrity of the epithelial barrier. On this basis, the central event of Hla is the formation of pores, rapidly changing the ion balance of the cell membrane (such as Ca²+ and K+ efflux) in a short period of time, and triggering a series of downstream reactions. This effect often exhibits the characteristics of “fast and strong”: when the pore load exceeds the repair threshold of the cell membrane, it synchronously amplifies phenotypes such as activation of inflammasomes, cell death, and instability of barrier function. While β-hemolysin (Hlb) centers on the activity of neutral sphingomyelinase. It does not necessarily directly cause acute cell lysis but rather reshapes the membrane lipid microenvironment by hydrolyzing sphingomyelin, thereby modifying the activity of membrane proteinases, the stability of cell connections, and the tissue repair process.

In the infection process, during the early stage of infection, Hla can participate in immune regulation and remodeling of cell responses when expressed at a low level; Hlb is generally expressed at a low level or absent in most strains due to the integration of φSa3int, reducing additional membrane lipid disturbances and triggering inflammation during the initial adhesion and microecological adaptation stages of the bacteria. In the progression stage, an increase in Hla levels is more likely to be associated with immune imbalance, inflammation amplification, and barrier damage; Hlb may be restored under stress induction and regulate the stability of the biofilm, chemotactic signal inhibition, or the regulation of the endothelium–platelet axis, increasing the probability and tolerance of bacterial colonization in the local area. In the later stage of infection, the acute barrier disruption caused by Hla can provide a pathway for bacterial dissemination; the anti-angiogenic effect of Hlb and the chronic inflammation maintenance related to membrane lipid remodeling may delay repair, making the lesion more difficult to return to a stable state.

In terms of functional positioning, Hla directly affects “whether acute injuries can be rapidly amplified”, while Hlb significantly influences “whether infections are prone to persist and whether colonization is stable”. This does not mean that the two can be simply classified as “acute factors” and “chronic factors” in clinical practice, because the microenvironment differences in different tissues (skin, nasal cavity, endocardium, lungs, etc.) will change toxin output and host thresholds. However, considering them as two different pathogenic modules helps explain why the same pathogen can present a broad spectrum of phenotypes ranging from asymptomatic carriage to severe invasion. In conclusion, understanding the mechanism of the virulence effects caused by Hla and Hlb is helpful for us to find effective targets to combat *S. aureus* infections.

## References

[1] Edwards AM Massey RC . How does Staphylococcus aureus escape the bloodstream? Trends Microbiol. (2011) 19:184–90. doi: 10.1007/978-1-4684-0243-8_2. PMID: 21227700

[2] Gardete S Tomasz A . Mechanisms of vancomycin resistance in Staphylococcus aureus. J Clin Invest. (2014) 124:2836–40. doi: 10.1172/jci68834. PMID: 24983424 PMC4071404

[3] Kebaier C Chamberland RR Allen IC Gao X Broglie PM Hall JD . Staphylococcus aureus α-hemolysin mediates virulence in a murine model of severe pneumonia through activation of the NLRP3 inflammasome. J Infect Dis. (2012) 205:807–17. doi: 10.1093/infdis/jir846 PMC3274379 22279123

[4] Widaa A Claro T Foster TJ O'Brien FJ Kerrigan SW . Staphylococcus aureus protein A plays a critical role in mediating bone destruction and bone loss in osteomyelitis. PloS One. (2012) 7:e40586. doi: 10.1371/journal.pone.0040586 22792377 PMC3394727

[5] Tong SY Davis JS Eichenberger E Holland TL Fowler VG Jr . Staphylococcus aureus infections: epidemiology, pathophysiology, clinical manifestations, and management. Clin Microbiol Rev. (2015) 28:603–61. doi: 10.1128/CMR.00134-14 PMC4451395 26016486

[6] Lakhundi S Zhang K . Methicillin-resistant Staphylococcus aureus: Molecular characterization, evolution, and epidemiology. Clin Microbiol Rev. (2018) 31:e00020-18. doi: 10.1128/cmr.00020-18. PMID: 30209034 PMC6148192

[7] Tenover FC Biddle JW Lancaster MV . Increasing resistance to vancomycin and other glycopeptides in Staphylococcus aureus. Emerging Infect Dis. (2001) 7:327–32. doi: 10.3201/eid0702.010237. PMID: 11294734 PMC2631729

[8] Zhu Z Hu Z Li S Fang R Ono HK Hu D-L . Molecular characteristics and pathogenicity of Staphylococcus aureus exotoxins. Int J Mol Sci. (2023) 25:395. doi: 10.3390/ijms25010395. PMID: 38203566 PMC10778951

[9] Jayasinghe L Miles G Bayley H . Role of the amino latch of staphylococcal alpha-hemolysin in pore formation: a co-operative interaction between the N terminus and position 217. J Biol Chem. (2006) 281:2195–204. doi: 10.1074/jbc.M510841200 16227199

[10] Song L Hobaugh MR Shustak C Cheley S Bayley H Gouaux JE . Structure of staphylococcal alpha-hemolysin, a heptameric transmembrane pore. Science. (1996) 274:1859–66. doi: 10.1126/science.274.5294.1859. PMID: 8943190

[11] Montoya M Gouaux E . Beta-barrel membrane protein folding and structure viewed through the lens of alpha-hemolysin. Biochim Biophys Acta. (2003) 1609:19–27. doi: 10.1016/s0005-2736(02)00663-6. PMID: 12507754

[12] Dal Peraro M Van Der Goot FG . Pore-forming toxins: ancient, but never really out of fashion. Nat Rev Microbiol. (2016) 14:77–92. doi: 10.1038/nrmicro.2015.3. PMID: 26639780

[13] Galdiero S Gouaux E . High resolution crystallographic studies of alpha-hemolysin-phospholipid complexes define heptamer-lipid head group interactions: implication for understanding protein-lipid interactions. Protein Science: A Publ Protein Soc. (2004) 13:1503–11. doi: 10.1110/ps.03561104 PMC2279993 15152085

[14] Valeva A Hellmann N Walev I Strand D Plate M Boukhallouk F . Evidence that clustered phosphocholine head groups serve as sites for binding and assembly of an oligomeric protein pore. J Biol Chem. (2006) 281:26014–21. doi: 10.1074/jbc.m601960200. PMID: 16829693

[15] Simons K Ehehalt R . Cholesterol, lipid rafts, and disease. J Clin Invest. (2002) 110:597–603. doi: 10.1172/jci0216390. PMID: 12208858 PMC151114

[16] Kulma M Hereć M Grudziński W Anderluh G Gruszecki WI Kwiatkowska K . Sphingomyelin-rich domains are sites of lysenin oligomerization: implications for raft studies. Biochim Biophys Acta. (2010) 1798:471–81. doi: 10.1016/j.bbamem.2009.12.004. PMID: 20018171

[17] Dreymueller D Uhlig S Ludwig A . ADAM-family metalloproteinases in lung inflammation: potential therapeutic targets. Am J Physiol Lung Cell Mol Physiol. (2015) 308:L325–43. doi: 10.1152/ajplung.00294.2014. PMID: 25480335

[18] Saftig P Lichtenthaler SF . The alpha secretase ADAM10: A metalloprotease with multiple functions in the brain. Prog Neurobiol. (2015) 135:1–20. doi: 10.1016/j.pneurobio.2015.10.003. PMID: 26522965

[19] Wilke GA Bubeck Wardenburg J . Role of a disintegrin and metalloprotease 10 in Staphylococcus aureus alpha-hemolysin-mediated cellular injury. Proc Natl Acad Sci USA. (2010) 107:13473–8. doi: 10.1073/pnas.1001815107. PMID: 20624979 PMC2922128

[20] Tosetti F Alessio M Poggi A Zocchi MR . ADAM10 site-dependent biology: Keeping control of a pervasive protease. Int J Mol Sci. (2021) 22:4969. doi: 10.3390/ijms22094969. PMID: 34067041 PMC8124674

[21] Shah J Rouaud F Guerrera D Vasileva E Popov LM Kelley WL . A dock-and-lock mechanism clusters ADAM10 at cell-cell junctions to promote α-toxin cytotoxicity. Cell Rep. (2018) 25:2132–47.e7. doi: 10.1016/j.celrep.2018.10.088. PMID: 30463011

[22] Olaniyi RO Pancotto L Grimaldi L Bagnoli F . Deciphering the pathological role of staphylococcal α-toxin and Panton-Valentine leukocidin using a novel ex vivo human skin model. Front Immunol. (2018) 9:951. doi: 10.3389/fimmu.2018.00951. PMID: 29867940 PMC5953321

[23] Wang K Liao J Yuan Y Chen Z Gou Q Jing H . Alpha hemolysin enhances the immune response by modulating dendritic cell differentiation via ADAM10-Notch signaling. Signal Transduct Target Ther. (2025) 10:334. doi: 10.1038/s41392-025-02432-3. PMID: 41062455 PMC12508477

[24] Koziel J Chmiest D Bryzek D Kmiecik K Mizgalska D Maciag-Gudowska A . The Janus face of α-toxin: a potent mediator of cytoprotection in staphylococci-infected macrophages. J Innate Immun. (2015) 7:187–98. doi: 10.1159/000368048. PMID: 25358860 PMC4348342

[25] Kitur K Parker D Nieto P Ahn DSA Cohen TS Chung S . Toxin-induced necroptosis is a major mechanism of Staphylococcus aureus lung damage. PloS Pathog. (2015) 11:e1004820. doi: 10.1371/journal.ppat.1004820. PMID: 25880560 PMC4399879

[26] Ezekwe EA Weng C Duncan JA . ADAM10 cell surface expression but not activity is critical for Staphylococcus aureus α-hemolysin-mediated activation of the NLRP3 inflammasome in human monocytes. Toxins. (2016) 8:95. doi: 10.3390/toxins8040095. PMID: 27043625 PMC4848622

[27] Craven RR Gao X Allen IC Gris D Bubeck Wardenburg J McElvania-Tekippe E . Staphylococcus aureus alpha-hemolysin activates the NLRP3-inflammasome in human and mouse monocytic cells. PloS One. (2009) 4:e7446. doi: 10.1371/journal.pone.0007446 19826485 PMC2758589

[28] Agostini L Martinon F Burns K McDermott MF Hawkins PN Tschopp J . NALP3 forms an IL-1beta-processing inflammasome with increased activity in Muckle-Wells autoinflammatory disorder. Immunity. (2004) 20:319–25. doi: 10.1016/s1074-7613(04)00046-9. PMID: 15030775

[29] Scherr TD Hanke ML Huang O James DBA Horswill AR Bayles KW . Staphylococcus aureus biofilms induce macrophage dysfunction through leukocidin AB and alpha-toxin. mBio. (2015) 6:e01021-15. doi: 10.1128/mBio.01021-15 26307164 PMC4550693

[30] Cohen TS Hilliard JJ Jones-Nelson O Keller AE O'Day T Tkaczyk C . Staphylococcus aureus α toxin potentiates opportunistic bacterial lung infections. Sci Transl Med. (2016) 8:329ra31. doi: 10.1126/scitranslmed.aad9922 26962155

[31] Cohen TS Boland ML Boland BB Takahashi V Tovchigrechko A Lee Y . S. aureus evades macrophage killing through NLRP3-dependent effects on mitochondrial trafficking. Cell Rep. (2018) 22:2431–41. doi: 10.1016/j.celrep.2018.02.027 PMC7160668 29490278

[32] Razooqi Z Bao K Yabrag A Ullah N Sitaram RT Lindholm M . Filifactor alocis FtxA blocks inflammation and apoptosis pathways in monocytic cells. Front Cell Infect Microbiol. (2026) 16:1745721. doi: 10.3389/fcimb.2026.1745721. PMID: 41947787 PMC13050831

[33] Bonifacius A Goldmann O Floess S Holtfreter S Robert PA Nordengrün M . Staphylococcus aureus alpha-toxin limits type 1 while fostering type 3 immune responses. Front Immunol. (2020) 11:1579. doi: 10.3389/fimmu.2020.01579 32849537 PMC7427519

[34] Choi SJ Kim M-H Jeon J Kim OY Choi Y Seo J . Active immunization with extracellular vesicles derived from Staphylococcus aureus effectively protects against staphylococcal lung infections, mainly via Th1 cell-mediated immunity. PloS One. (2015) 10:e0136021. doi: 10.1371/journal.pone.0136021. PMID: 26333035 PMC4558092

[35] Zhang F LeDue O Jun M Goulart C Malley R Lu Y-J . Protection against Staphylococcus aureus colonization and infection by B- and T-cell-mediated mechanisms. mBio. (2018) 9:e01949-18. doi: 10.1109/apsipaasc58517.2023.10317351. PMID: 30327437 PMC6191547

[36] Archer NK Harro JM Shirtliff ME . Clearance of Staphylococcus aureus nasal carriage is T cell dependent and mediated through interleukin-17A expression and neutrophil influx. Infect Immun. (2013) 81:2070–5. doi: 10.1038/bjc.1975.118. PMID: 23529621 PMC3676016

[37] Greenberg JA Hrusch CL Jaffery MR David MZ Daum RS Hall JB . Distinct T-helper cell responses to Staphylococcus aureus bacteremia reflect immunologic comorbidities and correlate with mortality. Crit Care (London England). (2018) 22:107. doi: 10.1186/s13054-018-2025-x. PMID: 29695270 PMC5916828

[38] Blümel E Munir Ahmad S Nastasi C Willerslev-Olsen A Gluud M Fredholm S . Staphylococcus aureus alpha-toxin inhibits CD8(+) T cell-mediated killing of cancer cells in cutaneous T-cell lymphoma. Oncoimmunology. (2020) 9:1751561. doi: 10.1080/2162402X.2020.1751561 32363124 PMC7185203

[39] Zhang Y Zhao Z Han P Qi J Zhang M Wang L . α-hemolysin targets LGALS3 (galectin 3) to promote intracellular survival of Staphylococcus aureus via lysosomal disruption and autophagy inhibition. Autophagy. (2026), 1–19. doi: 10.1080/15548627.2026.2642331 PMC13285668 41787826

[40] Virreira Winter S Zychlinsky A Bardoel BW . Genome-wide CRISPR screen reveals novel host factors required for Staphylococcus aureus α-hemolysin-mediated toxicity. Sci Rep. (2016) 6:24242. doi: 10.1038/srep24242. PMID: 27066838 PMC4828653

[41] Ziesemer S Möller N Nitsch A Müller C Beule AG Hildebrandt J-P . Sphingomyelin depletion from plasma membranes of human airway epithelial cells completely abrogates the deleterious actions of S. aureus alpha-toxin. Toxins (Basel). (2019) 11:126. doi: 10.3390/toxins11020126. PMID: 30791542 PMC6409578

[42] Krones D Rühling M Becker KA Kunz TC Sehl C Paprotka K . Staphylococcus aureus α-toxin induces acid sphingomyelinase release from a human endothelial cell line. Front Microbiol. (2021) 12:694489. doi: 10.3389/fmicb.2021.694489 34394034 PMC8358437

[43] Idone V Tam C Goss JW Toomre D Pypaert M Andrews NW . Repair of injured plasma membrane by rapid Ca2+-dependent endocytosis. J Cell Biol. (2008) 180:905–14. doi: 10.1083/jcb.200708010. PMID: 18316410 PMC2265401

[44] Reddy A Caler EV Andrews NW . Plasma membrane repair is mediated by Ca(2+)-regulated exocytosis of lysosomes. Cell. (2001) 106:157–69. doi: 10.1016/s0092-8674(01)00421-4. PMID: 11511344

[45] McNeil PL Miyake K Vogel SS . The endomembrane requirement for cell surface repair. PNAS. (2003) 100:4592–7. doi: 10.1073/pnas.0736739100. PMID: 12672953 PMC153600

[46] Inoshima N Wang Y Bubeck Wardenburg J . Genetic requirement for ADAM10 in severe Staphylococcus aureus skin infection. J Invest Dermatol. (2012) 132:1513–6. doi: 10.1038/jid.2011.462. PMID: 22377761 PMC3326222

[47] Solanas G Cortina C Sevillano M Batlle E . Cleavage of E-cadherin by ADAM10 mediates epithelial cell sorting downstream of EphB signalling. Nat Cell Biol. (2011) 13:1100–7. doi: 10.1038/ncb2298. PMID: 21804545

[48] Heinrich M Wickel M Schneider-Brachert W Sandberg C Gahr J Schwandner R . Cathepsin D targeted by acid sphingomyelinase-derived ceramide. EMBO J. (1999) 18:5252–63. doi: 10.1093/emboj/18.19.5252. PMID: 10508159 PMC1171596

[49] Ma J Gulbins E Edwards MJ Caldwell CC Fraunholz M Becker KA . Staphylococcus aureus α-toxin induces inflammatory cytokines via lysosomal acid sphingomyelinase and ceramides. Cell Physiol Biochemistry: Int J Exp Cell Physiology Biochemistry Pharmacol. (2017) 43:2170–84. doi: 10.1159/000484296 29069651

[50] Becker KA Fahsel B Kemper H Mayeres J Li C Wilker B . Staphylococcus aureus alpha-toxin disrupts endothelial-cell tight junctions via acid sphingomyelinase and ceramide. Infect Immun. (2018) 86:e00606-17. doi: 10.1128/IAI.00606-17 29084896 PMC5736828

[51] Yang C Robledo-Avila FH Partida-Sanchez S Montgomery CP . α-hemolysin-mediated endothelial injury contributes to the development of Staphylococcus aureus-induced dermonecrosis. Infect Immun. (2024) 92:e0013324. doi: 10.1128/iai.00133-24 38953668 PMC11320951

[52] Miller LS O'Connell RM Gutierrez MA Pietras EM Shahangian A Gross CE . MyD88 mediates neutrophil recruitment initiated by IL-1R but not TLR2 activation in immunity against Staphylococcus aureus. Immunity. (2006) 24:79–91. doi: 10.1016/j.immuni.2005.11.011. PMID: 16413925

[53] Castleman MJ Febbraio M Hall PR . CD36 is essential for regulation of the host innate response to Staphylococcus aureus α-toxin-mediated dermonecrosis. J Immunol (Baltimore Md: 1950). (2015) 195:2294–302. doi: 10.4049/jimmunol.1500500. PMID: 26223653 PMC4546912

[54] Bartlett AH Foster TJ Hayashida A Park PW . Alpha-toxin facilitates the generation of CXC chemokine gradients and stimulates neutrophil homing in Staphylococcus aureus pneumonia. J Infect Dis. (2008) 198:1529–35. doi: 10.1086/592758. PMID: 18823272 PMC12822823

[55] Weiss SJ . Tissue destruction by neutrophils. N Engl J Med. (1989) 320:365–76. doi: 10.1056/nejm198902093200606. PMID: 2536474

[56] Serhan CN . Pro-resolving lipid mediators are leads for resolution physiology. Nature. (2014) 510:92–101. doi: 10.1038/nature13479. PMID: 24899309 PMC4263681

[57] Serhan CN Levy BD . Resolvins in inflammation: emergence of the pro-resolving superfamily of mediators. J Clin Invest. (2018) 128:2657–69. doi: 10.1172/jci97943. PMID: 29757195 PMC6025982

[58] Jordan PM Gerstmeier J Pace S Bilancia R Rao Z Börner F . Staphylococcus aureus-derived α-hemolysin evokes generation of specialized pro-resolving mediators promoting inflammation resolution. Cell Rep. (2020) 33:108247. doi: 10.1016/j.celrep.2020.108247 33053344 PMC7729929

[59] Zhang H Zheng Y Gao H Xu P Wang M Li A . Identification and characterization of Staphylococcus aureus strains with an incomplete hemolytic phenotype. Front Cell Infect Microbiol. (2016) 6:146. doi: 10.1007/978-1-0716-2639-9_44. PMID: 27917374 PMC5114236

[60] Johansson C Rautelin H Kaden R . Staphylococcus argenteus and Staphylococcus schweitzeri are cytotoxic to human cells *in vitro* due to high expression of alpha-hemolysin Hla. Virulence. (2019) 10:502–10. doi: 10.1080/21505594.2019.1620062 PMC6550535 31131704

[61] Projan SJ Kornblum J Kreiswirth B Moghazeh SL Eisner W Novick RP . Nucleotide sequence: the beta-hemolysin gene of Staphylococcus aureus. Nucleic Acids Res. (1989) 17:3305. doi: 10.1093/nar/17.8.3305 2726469 PMC317744

[62] Walev I Weller U Strauch S Foster T Bhakdi S . Selective killing of human monocytes and cytokine release provoked by sphingomyelinase (beta-toxin) of Staphylococcus aureus. Infect Immun. (1996) 64:2974–9. doi: 10.1128/iai.64.8.2974-2979.1996. PMID: 8757823 PMC174177

[63] Huseby M Shi K Brown CK Digre J Mengistu F Seo KS . Structure and biological activities of beta toxin from Staphylococcus aureus. J Bacteriol. (2007) 189:8719–26. doi: 10.1107/s0108767308088648. PMID: 17873030 PMC2168928

[64] Vandenesch F Lina G Henry T . Staphylococcus aureus hemolysins, bi-component leukocidins, and cytolytic peptides: a redundant arsenal of membrane-damaging virulence factors? Front Cell Infect Microbiol. (2012) 2:12. doi: 10.3389/fcimb.2012.00012 22919604 PMC3417661

[65] Tran PM Feiss M Kinney KJ Salgado-Pabón W . ϕSa3mw prophage as a molecular regulatory switch of Staphylococcus aureus β-toxin production. J Bacteriol. (2019) 201:e00766-18. doi: 10.1128/JB.00766-18 30962356 PMC6597384

[66] Katayama Y Baba T Sekine M Fukuda M Hiramatsu K . Beta-hemolysin promotes skin colonization by Staphylococcus aureus. J Bacteriol. (2013) 195:1194–203. doi: 10.1128/jb.01786-12. PMID: 23292775 PMC3592002

[67] Verkaik NJ Benard M Boelens HA de Vogel CP Nouwen JL Verbrugh HA . Immune evasion cluster-positive bacteriophages are highly prevalent among human Staphylococcus aureus strains, but they are not essential in the first stages of nasal colonization. Clin Microbiol Infection: Off Publ Eur Soc Clin Microbiol Infect Dis. (2011) 17:343–8. doi: 10.1111/j.1469-0691.2010.03227.x. PMID: 20370801

[68] Huseby MJ Kruse AC Digre J Kohler PL Vocke JA Mann EE . Beta toxin catalyzes formation of nucleoprotein matrix in staphylococcal biofilms. PNAS. (2010) 107:14407–12. doi: 10.1073/pnas.0911032107. PMID: 20660751 PMC2922554

[69] Salgado-Pabón W Herrera A Vu BG Stach CS Merriman JA Spaulding AR . Staphylococcus aureus β-toxin production is common in strains with the β-toxin gene inactivated by bacteriophage. J Infect Dis. (2014) 210:784–92. doi: 10.1093/infdis/jiu146 PMC4202305 24620023

[70] Herrera A Kulhankova K Sonkar VK Dayal S Klingelhutz AJ Salgado-Pabón W . Staphylococcal β-toxin modulates human aortic endothelial cell and platelet function through sphingomyelinase and biofilm ligase activities. mBio. (2017) 8:e00273-17. doi: 10.1128/mbio.00273-17. PMID: 28325766 PMC5362035

[71] Bussolino F Di Renzo MF Ziche M Bocchietto E Olivero M Naldini L . Hepatocyte growth factor is a potent angiogenic factor which stimulates endothelial cell motility and growth. J Cell Biol. (1992) 119:629–41. doi: 10.1083/jcb.119.3.629. PMID: 1383237 PMC2289675

[72] Moccia F Negri S Shekha M Faris P Guerra G . Endothelial Ca(2+) signaling, angiogenesis and vasculogenesis: just what it takes to make a blood vessel. Int J Mol Sci. (2019) 20:3962. doi: 10.3390/ijms20163962. PMID: 31416282 PMC6721072

[73] Dong F Zhang X Wold LE Ren Q Zhang Z Ren J . Endothelin-1 enhances oxidative stress, cell proliferation and reduces apoptosis in human umbilical vein endothelial cells: role of ETB receptor, NADPH oxidase and caveolin-1. Br J Pharmacol. (2005) 145:323–33. doi: 10.1038/sj.bjp.0706193. PMID: 15765100 PMC1576147

[74] Lawler J . Thrombospondin-1 as an endogenous inhibitor of angiogenesis and tumor growth. J Cell Mol Med. (2002) 6:1–12. doi: 10.1111/j.1582-4934.2002.tb00307.x. PMID: 12003665 PMC6740251

[75] Cabral-Pacheco GA Garza-Veloz I Castruita-De La Rosa C Ramirez-Acuña JM Perez-Romero BA Guerrero-Rodriguez JF . The roles of matrix metalloproteinases and their inhibitors in human diseases. Int J Mol Sci. (2020) 21:9739. doi: 10.3390/ijms21249739. PMID: 33419373 PMC7767220

[76] Tran PM Tang SS Salgado-Pabón W . Staphylococcus aureus β-toxin exerts anti-angiogenic effects by inhibiting re-endothelialization and neovessel formation. Front Microbiol. (2022) 13:840236. doi: 10.3389/fmicb.2022.840236 35185854 PMC8851161

[77] Jia Y Guan Z Liu C Huang M Li J Feng J . Staphylococcus aureus β-hemolysin causes skin inflammation by acting as an agonist of epidermal growth factor receptor. Microbiol Spectr. (2024) 12:e0222723. doi: 10.1128/spectrum.02227-23 38059627 PMC10783061

[78] Guan Z Liu Y Liu C Wang HT Feng JN Yang G . Staphylococcus aureus β-hemolysin up-regulates the expression of IFN-γ by human CD56(bright) NK cells. Front Cell Infect Microbiol. (2021) 11:658141. doi: 10.3389/fcimb.2021.658141 33854984 PMC8039520

[79] Klunker S Trautmann A Akdis M Verhagen J Schmid-Grendelmeier P Blaser K . A second step of chemotaxis after transendothelial migration: keratinocytes undergoing apoptosis release IFN-gamma-inducible protein 10, monokine induced by IFN-gamma, and IFN-gamma-inducible alpha-chemoattractant for T cell chemotaxis toward epidermis in atopic dermatitis. J Immunol (Baltimore Md: 1950). (2003) 171:1078–84. doi: 10.1159/000053720. PMID: 12847282

[80] Antonelli A Ferrari SM Giuggioli D Ferrannini E Ferri C Fallahi P . Chemokine (C-X-C motif) ligand (CXCL)10 in autoimmune diseases. Autoimmun Rev. (2014) 13:272–80. doi: 10.1016/j.autrev.2013.10.010. PMID: 24189283

[81] Di Bari F . Atopic dermatitis and alpha-chemokines. Clin Ter. (2015) 166:e182–7. doi: 10.7417/CT.2015.1852 26152630

[82] Lin T Li Q Jin D Liu W Tang C Zhang X . Investigation of virulence genes of Staphylococcus aureus isolated from sterile body fluid samples and their correlation with clinical symptoms and outcomes. Can J Infect Dis Med Microbiol. (2021) 2021:5354747. doi: 10.1155/2021/5354747. PMID: 34987680 PMC8720599

[83] Orellana P Andrade CF Ginestre M Orellana MR . Virulent strains of Staphylococcus aureus isolatedfrom healthcare personnel in a hospital center. Genet Mol Res. (2024) 23(3):gmr2346. doi: 10.4238/gmr2346

[84] Liu L Zhuang H Wang Y Tu Y Yu Y Chen Y . β-hemolysin, not agrA mutation, inhibits the hemolysis of α-hemolysin in Staphylococcus aureus laboratory and clinical strains. mSphere. (2024) 9:e0067323. doi: 10.1128/msphere.00673-23 38289073 PMC10900901

[85] Tang W Liu Y Li X Leng G Gao J Wang Y . Microbiological characteristics of clinically isolated Staphylococcus aureus with different hemolytic phenotypes in China. Infect Drug Resist. (2024) 17:3273–87. doi: 10.2147/idr.s466416. PMID: 39104458 PMC11299731

[86] Li Q Chen N Liu C Zhao Z Huang M Li J . Staphylococcus aureus β-hemolysin impairs oxygen transport without causing hemolysis. Virulence. (2025) 16:2490208. doi: 10.1080/21505594.2025.2490208 40202859 PMC11988224

[87] Shekhar A Di Lucrezia R Jerye K Korotkov VS Harmrolfs K Rox K . Highly potent quinoxalinediones inhibit α-hemolysin and ameliorate Staphylococcus aureus lung infections. Cell Host Microbe. (2025) 33:560–572.e21. doi: 10.1016/j.chom.2025.03.006 40168998

[88] Sakari M Laisi A Pulliainen AT . Exotoxin-targeted drug modalities as antibiotic alternatives. ACS Infect Dis. (2022) 8:433–56. doi: 10.1021/acsinfecdis.1c00296. PMID: 35099182 PMC8922280

[89] Tomaszewski KL Blanchard M Olaniyi R Brenton HR Hayes S Fatma F . Enhanced Staphylococcus aureus protection by uncoupling of the α-toxin-ADAM10 interaction during murine neonatal vaccination. Nat Commun. (2024) 15:8702. doi: 10.1016/j.solener.2018.05.018. PMID: 39379345 PMC11461939

[90] Hao L Zhou J Yang H He C Shu W Song H . Anti-virulence potential of iclaprim, a novel folic acid synthesis inhibitor, against Staphylococcus aureus. Appl Microbiol Biotechnol. (2024) 108:432. doi: 10.1007/s00253-024-13268-2. PMID: 39102054 PMC11300511

[91] Wang G Wen J Tian Z Zhou H Peng X Zhang P . Piceatannol and its analogues alleviate Staphylococcus aureus pathogenesis by targeting β-lactamase biofilms and α-hemolysin. Sci Rep. (2025) 15:5551. doi: 10.1038/s41598-025-89654-1. PMID: 39952994 PMC11828952

